# Rhabdomyolysis as a Presentation of 2019 Novel Coronavirus Disease

**DOI:** 10.7759/cureus.7561

**Published:** 2020-04-06

**Authors:** Kulachanya Suwanwongse, Nehad Shabarek

**Affiliations:** 1 Internal Medicine, Lincoln Medical Center, New York City, USA

**Keywords:** covid-19, corona virus, novel corona virus, covid-2019, ards, case report, rhabdomyolysis

## Abstract

An emerging viral infection is a global public health challenge. The development of modern, fast, and extensive transportation makes the outbreak hard to contain. Everyone is at risk, and the outbreak can rapidly turn into a pandemic crisis, like what we are currently facing for the 2019 novel coronavirus disease (COVID-19). Prompt diagnosis of the case is required to improve patients’ prognosis and control of the outbreak. The common manifestations of COVID-19 include fever, cough, dyspnea, and malaise. However, patients may present with atypical symptoms that pose a diagnostic challenge. We report the first case of an elderly male who presented with rhabdomyolysis and later was diagnosed with COVID-19. Clinicians should be aware that rhabdomyolysis can be an initial presentation of COVID-19 or can occur at any time during the disease course. Patients with rhabdomyolysis should receive aggressive fluid administration to prevent acute kidney injury (AKI). However, COVID-19 patients are at risk of worsening oxygenation and acute hypoxemic respiratory failure from fluid overload. Therefore, cautious fluid administration is needed in COVID-19 patients with rhabdomyolysis.

## Introduction

2019 novel coronavirus disease (COVID-19) is a global public health crisis, causing alarming numbers of morbidity and mortality. Prompt diagnosis of the disease is required to improve patients’ outcomes and control the pandemic outbreak. The common clinical manifestations of COVID-19 are fever, cough, myalgia, and fatigue; while headache, hemoptysis, and gastrointestinal symptoms are less common [1]. Recently, there was a report of a COVID-19 patient from Wuhan, China, who developed rhabdomyolysis during hospitalization [2]. Herein, we report the first case of an elderly male who presented with rhabdomyolysis and later was diagnosed with COVID-19.

## Case presentation

**Figure 1:**
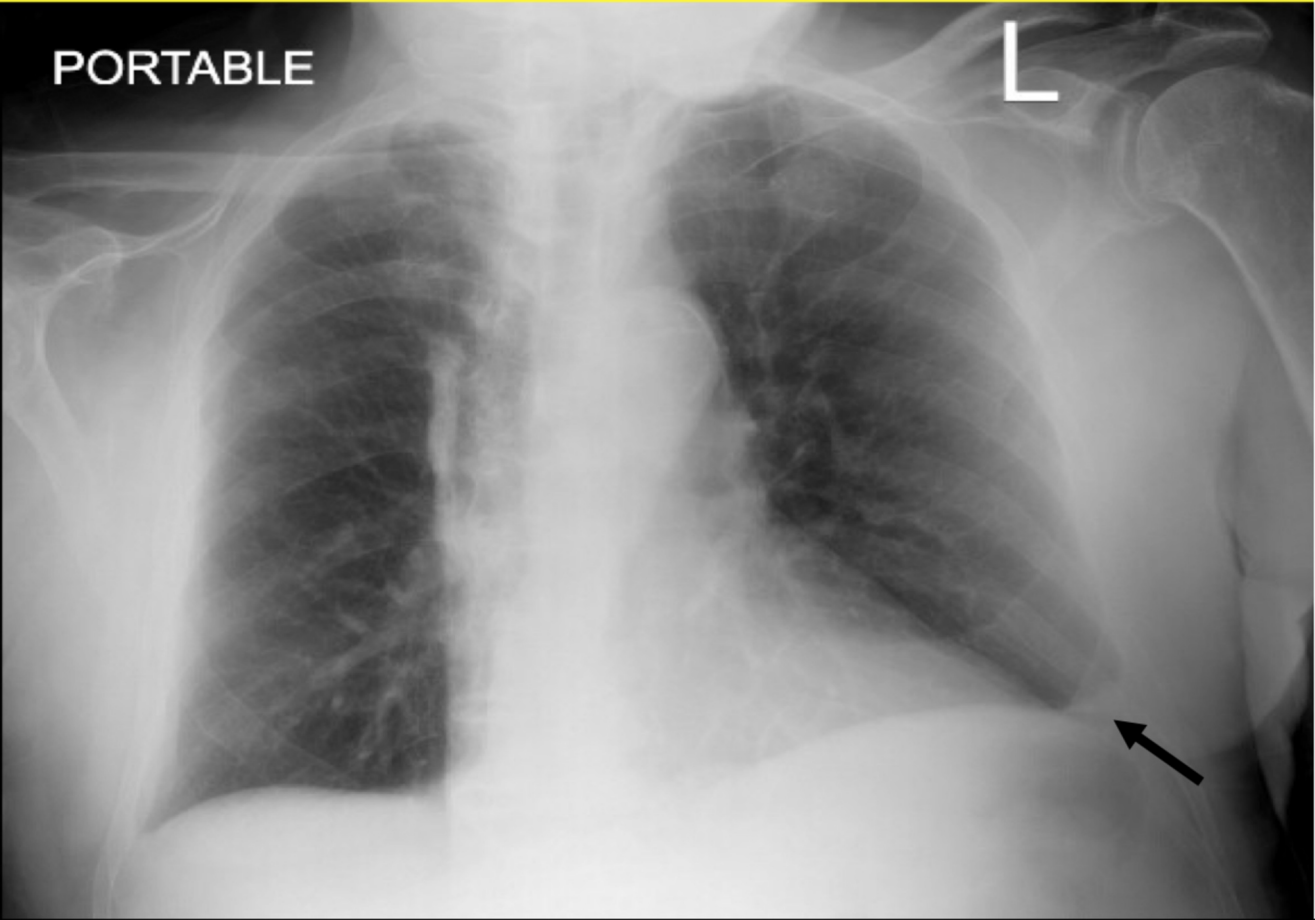
Chest X-ray (CXR) showed left small pleural effusion

An 88-year-old man presented to the emergency department with acute onset bilateral thighs weakness and pain. His home health attendance found him sat on the toilet and could not stand up due to the pain and weakness of both thighs. He had a past medical history of hypertension, chronic kidney disease, heart failure with reduced ejection fraction, benign prostatic hypertrophy, bilateral knees osteoarthritis, and mild cognitive impairment. His medications include simvastatin, donepezil, furosemide, losartan, metoprolol succinate, and tamsulosin, which were not changed since the past year. He took simvastatin for several years without any complications. He denied fever but admitted having some dry cough. He denied any other respiratory or gastrointestinal tract symptoms. On initial evaluation, he had a low-grade fever with a temperature of 100.2 Fahrenheit, and tachypnea with a respiratory rate of 22. Oxygen saturation was 94 percent on room air. His lungs exam revealed mild crackles in both basal lungs fields. His chest X-ray (CXR) showed blunting of the left costophrenic angle, indicating a small pleural effusion, which did not change compared to the CXR from the previous month (Figure 1). His pelvis X-ray was normal.

**Figure 2:**
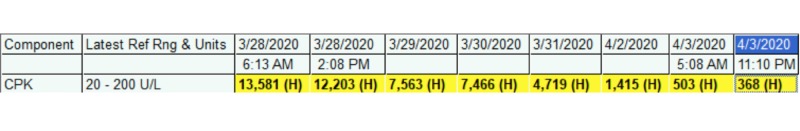
The trend of creatine phosphokinase (CPK)

His blood work was remarkable for the marked elevation of creatine phosphokinase (CPK) at 13,581 units per liters (U/L). Lactate dehydrogenase (LDH) was 364 U/L (normal range: 120-250 U/L). His creatinine was 1.16 milligrams per deciliter (mg/dl), which did not increase from his baseline. His urine analysis showed a presence of blood on a urine dipstick test with absent red blood cells. The COVID-19 polymerase chain reaction test from the nasopharyngeal swab was sent due to the presence of fever and cough and returned positive. The flu test was negative. He received a total of 2 L bolus of intravenous crystalloids but led to a mild exacerbation of his congestive heart failure, which was resolved after giving intravenous furosemide. Hydroxychloroquine was given for five days. His symptoms were improved. He reported less pain and fatigue, and his CPK was gradually decreased, as demonstrated in Figure 2. However, he developed acute kidney injury on hospital day 7 with a creatinine of 1.38 mg/dl. Small boluses of intravenous crystalloids (total of 1 L) were administered and his creatinine decreased to 1.09 mg/dl on the next day.

## Discussion

Rhabdomyolysis is a life-threatening condition caused by skeletal muscle damage from various etiologies. Viral-associated rhabdomyolysis is a common cause of rhabdomyolysis in pediatric patients, but it rarely occurs in adults and the elderly [3]. Influenza is the most common virus associated with rhabdomyolysis. Other viruses that may cause rhabdomyolysis includes human immunodeficiency virus (HIV), enteroviruses, Epstein-Barr virus (EBV), cytomegalovirus (CMV), adenovirus, herpes simplex virus (HSV), and varicella virus [4].

Recently, a case of COVID-19-induced rhabdomyolysis was reported from Wuhan, China, in which the patient developed rhabdomyolysis on hospital day 9. We reported the first case of a COVID-19 patient who presented with symptoms of rhabdomyolysis, including muscles pain and weakness.

There are several possible hypotheses explaining the pathogenesis of viral-induced rhabdomyolysis. Firstly, direct viral invasion can lead to rhabdomyolysis [5]. Secondly, a robust immune response to viruses resulting in cytokine storms and damaging muscle tissues. Thirdly, circulating viral toxins may directly destroy muscle cell membranes [5]. However, the mechanism of COVID-19-induced rhabdomyolysis has not yet been studied. We proposed that the excessive immune response and cytokine storms which often seen in COVID-19 patients at least partly contribute to rhabdomyolysis.

Our case report has limitations. We did not investigate for some common viral causes of rhabdomyolysis, including HIV, CMV, and EBV, which might be co-infected with COVID-19. In addition, our patient may have rhabdomyolysis from other causes and COVID-19 might be an incidental finding, given a currently high incidence of COVID-19 in New York City.

Myalgia and weakness are common symptoms of COVID-19, but clinicians should be aware that rhabdomyolysis may also occur, particularly when patients report focal muscle pain and weakness. CPK levels should be obtained in suspicious cases. In general, patients with rhabdomyolysis should receive aggressive fluid administration to prevent acute kidney injury (AKI). However, cautious fluid administration is needed in patients with heart failure as it may result in heart failure exacerbation as seen in our case. Serial creatinine is required in patients with rhabdomyolysis to early detect and promptly manage of AKI to prevent renal failure.

Management of rhabdomyolysis in COVID-19 patients is challenging. Aggressive fluid administration may worsen oxygenation especially in patients with acute respiratory distress syndrome (ARDS). We suggested giving small boluses of intravenous fluid (i.e., 250 ml) to the patients with close clinical observation, continuous monitor of oxygen saturation, and serial blood works for creatinine and CPK. Repeat small boluses of fluid should be given if patients' creatinine rises.

## Conclusions

Rhabdomyolysis can be an initial presentation of COVID-19 or may present at any time of the disease course. Clinicians should have high suspicion for rhabdomyolysis in COVID-19 patients with localized muscle pain or weakness. Prompt recognition and appropriate treatment improve patients’ outcomes.
